# Differential effects of hyaluronan synthase 3 deficiency after acute vs chronic liver injury in mice

**DOI:** 10.1186/s13069-016-0041-5

**Published:** 2016-03-31

**Authors:** Jennifer M. McCracken, Lu Jiang, Krutika T. Deshpande, Maura F. O’Neil, Michele T. Pritchard

**Affiliations:** Department of Pharmacology, Toxicology and Therapeutics, University of Kansas Medical Center, Kansas City, KS 66160 USA; Department of Pathology, University of Kansas Medical Center, Kansas City, KS 66160 USA

**Keywords:** Fibrosis, Hyaluronan, Inflammation, Liver, Matrix metalloproteinase 13

## Abstract

**Background:**

Hyaluronan (HA) is a ubiquitous extracellular matrix (ECM) glycosaminoglycan synthesized by three different enzymes, hyaluronan synthase (HAS)1, 2, and 3. HA synthesis mediated by HAS3 promotes inflammation and is pathogenic in animal models of human lung and intestinal disease. Liver fibrosis is a common endpoint to chronic liver injury and inflammation for which there is no cure. Although plasma HA is a commonly used biomarker for liver disease, if and how HA contributes to disease pathogenesis remains unclear. Here, we tested the hypothesis that HA synthesized by HAS3 enhances inflammation and fibrosis. To test this hypothesis, we exposed wild-type or *Has3−/−* mice to carbon tetrachloride (CCl_4_) once (acute) or ten (chronic) times.

**Results:**

HAS3-deficient mice exhibited increased hepatic injury and inflammatory chemokine production 48 h after acute CCl_4_; this was associated with a threefold reduction in plasma HA levels and alterations in the proportions of specific molecular weight HA polymer pools. Hepatic accumulation of fibrosis-associated transcripts was also greater in livers from HAS3-deficient mice compared to controls after acute CCl_4_ exposure. Surprisingly, fibrosis was not different between genotypes. Hepatic matrix metalloproteinase (MMP)13 mRNA and MMP13 activity was greater in livers from Has3-null mice after chronic CCl_4_; this was prevented by a MMP13-specific inhibitor. Collectively, these data suggest that Has3, or more likely HA produced by HAS3, limits hepatic inflammation after acute injury and attenuates MMP13-mediated matrix metabolism after chronic injury.

**Conclusions:**

These data suggest that HA should be investigated further as a novel therapeutic target for acute and chronic liver disease.

**Electronic supplementary material:**

The online version of this article (doi:10.1186/s13069-016-0041-5) contains supplementary material, which is available to authorized users.

## Background

Liver fibrosis is the end result of chronic hepatic injury and inflammation coupled with incomplete tissue repair. The net effect of incomplete repair over several cycles of tissue injury is accumulation of extracellular matrix (ECM) proteins. When prolonged, excessive ECM accumulation impacts hepatic architecture and function. Several agents cause liver fibrosis including alcohol, obesity, viruses, congenital disorders, cholestasis, parasites, drugs, and toxins [1, 2]. Regardless of etiologic agent, the progression to liver fibrosis occurs in only a subset of patients and is affected by a host of additional factors including genetics, environment, behavior, and various comorbidities [3]. While removal of the etiologic agent can attenuate disease progression and even lead to fibrosis reversal in some patients, no pharmacologic strategy yet exists to “cure” advanced liver disease [4, 5]. Liver transplantation is the only therapeutic option for advanced liver disease not responsive to etiologic agent removal.

Hyaluronan (HA) is a non-sulfated, anionic glycosaminoglycan which consists of repeating N-acetylglucosamine and glucuronic acid disaccharide units [6]. Three HA synthases exist in mammals: (HAS)1, 2, and 3 [7]. They differ based on biosynthetic capacity, length of the HA polymer synthesized, induction profiles, and tissue expression levels [7–9]. Generally speaking, HA is synthesized as a high molecular weight (HMW) polymer by many cell types including fibroblasts and vascular endothelial cells [10, 11]. When tissues are injured, HA is synthesized and degraded and participates in the wound healing response [11]. HMW-HA (250–2000 kDa) is a major component of healthy articular joints, the vitreous humor of the eye, and the skin and when synthesized in these injured tissues, dampens inflammation [12]. HA is degraded by hyaluronidases and reactive oxygen species under normal circumstances and also when tissues are injured into a polydisperse population containing low molecular weight (LMW) fragments (<250 kDa) which exacerbate inflammation [12]. Persistence of LMW-HA in chronically wounded tissue contributes to disease pathogenesis in animal models of idiopathic pulmonary fibrosis [13]. Of the hyaluronan synthases, HAS3 makes the smallest HA polymers ranging from 100 to 1000 kDa, which includes polymers in the LMW range [8]. Consistently, published studies using animal models of human lung and intestine disease show that HA synthesized by HAS3 contributes to inflammation [14–16].

HA is used as a biomarker for liver disease severity; the more advanced the liver disease, the greater the amount of HA is found in the blood [17, 18]. This is likely due to a combination of increased HA synthesis by hepatic stellate cells and other cells [19, 20], as well as a reduced capacity for HA uptake by dysfunctional liver sinusoidal endothelial cells found in diseased liver [21]. Despite the relationship between plasma HA levels and liver disease and the pro-inflammatory role HA plays in other diseases, no studies have determined whether or not HA has a direct role in liver disease pathogenesis. In this study, we tested the hypothesis that HAS3, or HA produced by HAS3, contributes to liver inflammation and fibrosis by increasing the proportion of LMW-HA polymers relative to HMW-HA polymers. To test this hypothesis, we utilized the well-characterized carbon tetrachloride (CCl_4_)-induced liver injury and fibrosis model in wild-type mice and mice deficient in HAS3 [22]. Due to a lack of HA synthesized by HAS3, we predicted *Has3−/−* mice would exhibit reduced inflammation and fibrosis after CCl_4_ exposure, similar to the previously published studies [14–16]. Here, we provide evidence that HAS3 plays divergent roles depending upon whether liver injury is acute or chronic.

## Results

### Hepatic *Has* gene transcript accumulation and plasma HA levels in wild-type and *Has3−/−* mice

**Fig. 1:**
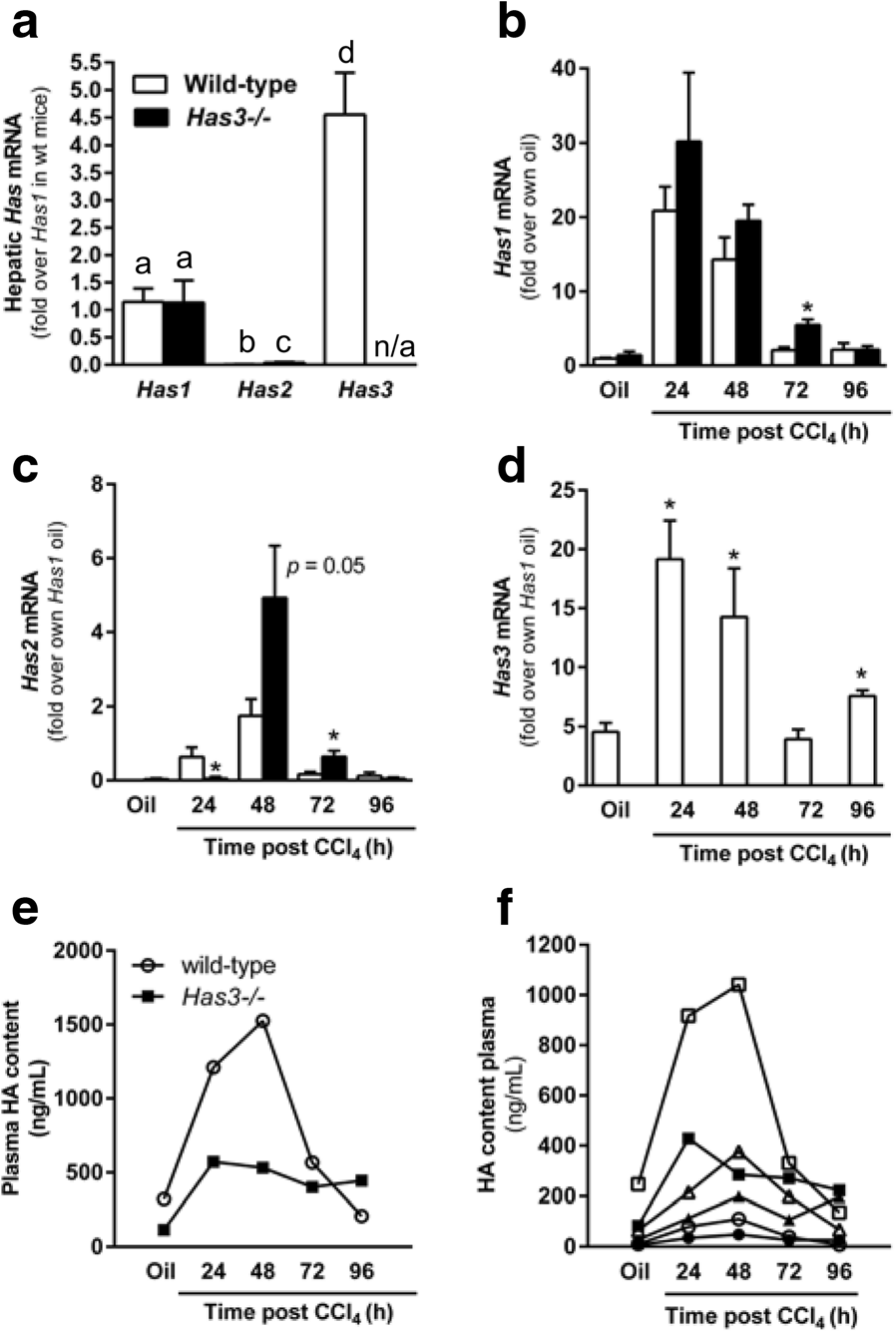
Hepatic *Has* enzymes and plasma HA levels in wild-type and *Has3−/−* mice. **a** Real-time PCR was used to measure basal levels of hepatic *Has* enzyme transcript accumulation in the wild-type and *Has3−/−* mice. Data were normalized to 18S and then expressed as fold change over wild-type hepatic *Has1* content. *Bars* with different *alphabetical superscripts* are significantly different from one another (*P* < 0.05). **b**–**d** The mice were exposed to CCl_4_ and euthanized 24, 48, 72, or 96 h later. Control animals received an olive oil (oil) injection. Real-time PCR was used to measure hepatic *Has1* (**b**), *Has2* (**c**), and *Has3* (**d**) in the livers from the wild-type and HAS3-deficient mice. *P < 0.05 when compared to the wild-type mice at the same time point. An ELISA-like assay (see the “Methods” section for details on this antibody-independent assay) was used to determine HA concentration in pooled plasma from the wild-type and *Has3−/−* mice either before (**e**) or after (**f**) HA fractionation using molecular weight cut-off columns. In **e** and **f**, the *open symbols* indicate wild-type mice while the *closed symbols* indicate *Has3−/−* mice. *Circles* indicate HA pools less than 100 kDa, *squares* indicate HA pools between 100 and 300 kDa, and *triangles* indicate HA pools greater than 300 kDa. Statistical analysis was not performed on these pooled samples. See Table 1 for additional details. *n/a* not applicable. *N* = 6–8 mice per experimental group

Real-time polymerase chain reaction (PCR) was used to determine the relative amounts of *Has1*, *2*, and *3* in the livers from wild-type and *Has3−/−* mice, at baseline. The accumulation of *Has1* transcripts was not different between genotypes (Fig. 1a). *Has3* transcripts were most abundant in the livers from the wild-type mice and were approximately fivefold more than Has1 transcripts. *Has2* transcripts were least abundant in both genotypes, between 1 and 5 % of total *Has1* levels. However, *Has2*, was 40 % greater in livers from *Has3−/−* mice relative to wild-type mice suggesting this enzyme may compensate, at least in part, for HAS3 deficiency at baseline.

Real-time PCR was again used to evaluate hepatic *Has* gene transcript levels after acute CCl_4_ exposure. In wild-type mice, *Has1* levels were increased 20-fold above baseline 24 h after CCl_4_ exposure and declined thereafter (Fig. 1b). In the *Has3−/−* mice, hepatic *Has1* transcripts also increased 24 h after CCl_4_ exposure, but this level was not different from that found in the wild-type mice (Fig. 1b). *Has1* transcripts were greater in the livers from the *Has3−/−* mice relative to the wild-type mice 72 h after CCl_4_ exposure. *Has2* transcripts peaked in the wild-type mice 48 h after CCl_4_ exposure, but only 1.8-fold above baseline (Fig. 1c). Unlike *Has2* transcripts in the wild-type mice, *Has2* transcripts were not induced in the *Has3−/−* mice until 48 h after CCl_4_ exposure (Fig. 1c). At this time point, *Has2* transcripts increased fivefold in the *Has3−/−* mice, but this increase was not significantly different than that found in the wild-type mice (*P* = 0.05, Fig. 1c). However, *Has2* transcripts were greater in the *Has3−/−* mice compared to the wild-type mice 72 h after CCl_4_ exposure (Fig. 1c). Finally, *Has3* transcripts were induced approximately 20-fold over baseline 24 and 48 h after CCl_4_ exposure in wild-type mice and returned to baseline 72 h after CCl_4_ and increased again 96 h after CCl_4_ (Fig. 1d). Taken together, acute CCl_4_ exposure induced all three *Has* transcripts in the liver; *Has1* and *Has3* were most robustly induced.

Using an enzyme-linked immunosorbent assay (ELISA)-like assay, we measured plasma HA levels in the wild-type and *Has3−/−* mice. The *Has3−/−* mice had reduced plasma HA levels when compared to the wild-type mice at baseline, 24 h, and 48 h after CCl_4_ exposure (Fig. 1e). HA levels were similar between genotypes at 72 h but greater in the *Has3−/−* mice 96 h after CCl_4_ exposure (Fig. 1e). These data suggested that other HAS enzymes did not completely compensate for reduced HA biosynthetic capacity found in the *Has3−/−* mice. Next, using specific molecular weight cut-off columns to fractionate plasma HA into three groups (<100 kDa, 100–300 kDa, and >300 kDa), we found that the wild-type mice had more HA in the 100–300 kDa molecular weight range, compared to the *Has3−/−* mice, while the absolute amounts of HA in the other fractions did not exhibit such large differences between genotypes (Fig. 1f). The majority of plasma HA was found in the 100–300 kDa fraction in the wild-type and *Has3−/−* mice (Fig. 1f and Table 1). However, relative to wild-type mice, the *Has3−/−* mice exhibited an increased percentage of the <100 kDa HA fraction, decreased percentage of the 100–300 kDa fraction, and increased percentage of the >300 kDa HA fraction at almost every time point after CCl_4_ exposure (Table 1). These data suggested that, in addition to a reduction in total HA level, HA molecular mass distribution was also perturbed in the *Has3−/−* mice, favoring lower molecular weight populations, relative to the wild-type mice.

**Table 1:** Total and fractionated plasma HA content in wild-type and *Has3−/−* mice after acute carbon tetrachloride exposure

Group	<100 kDa (ng/mL)	% of total	100–300 kDa (ng/mL)	% of total	>300 kDa (ng/mL)	% of total	Total (ng/mL)
Wild-type mice							
Oil	10	3	247	77	64	20	322
24 h	78	6	917	76	215	18	1210
48 h	109	7	1041	68	376	25	1526
72 h	38	7	330	58	199	35	567
96 h	7	3	135	65	66	32	208
*Has3−/−* mice							
Oil	6	5	82	71	27	23	115
24 h	34	3	428	75	112	20	574
48 h	48	9	285	54	199	37	532
72 h	26	7	271	67	106	26	403
96 h	26	6	223	50	196	44	445

### Liver injury and steatosis after acute CCl_4_ exposure

**Fig. 2:**
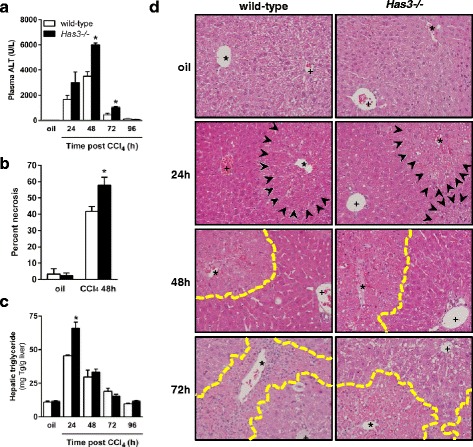
Liver injury and steatosis in wild-type and *Has3−/−* mice. The mice were exposed to CCl_4_ and euthanized 24, 48, 72, or 96 h later. Control animals received an olive oil (oil) injection. **a** Plasma ALT activity was determined using an enzymatic assay. **b** Histopathologic assessment of the percent necrosis at baseline and 48 h after CCl_4_ exposure. **c** Total hepatic triglycerides were measured using a biochemical assay. **d** Representative histology in the wild-type and *Has3−/−* mice (200×). *Asterisks* denote central veins, and *plus signs* denote the portal vein of the portal triad. The necrotic areas are outlined with *yellow dashed line* in the 48 and 72 h images, and the area of macrovesicular steatosis is identified by *black arrows* in the 24 h images. In every figure of this manuscript, *white bars* indicate wild-type mice while *black bars* indicate *Has3−/−* mice. *P < 0.05 compared between genotypes at the same time point. *N* = 4–7 mice per experimental group

The wild-type and *Has3−/−* mice were exposed to CCl_4_ and euthanized 24, 48, 72, and 96 h later. CCl_4_ induced liver injury in both strains of mice, but this injury was greater in the livers from the *Has3−/−* mice. Specifically, peak plasma alanine aminotransferase (ALT) activity, a measure of liver injury, was 41 % greater in the *Has3−/−* mice relative to the control mice 48 h after CCl_4_ exposure (Fig. 2a). Although plasma ALT activity was reduced 72 h after CCl_4_ in both strains, it remained greater in the *Has3−/−* mice (Fig. 2a). In support of these data, histopathological assessment revealed more necrosis in the livers from the *Has3−/−* mice compared to the wild-type mice (Fig. 2b). Hepatic triglyceride accumulation increased 24 h after CCl_4_ but was 31 % greater in the HAS3-deficient mice compared to the controls (Fig. 2c). Liver histology paralleled these two biochemical measures of the liver’s response to CCl_4_ in the wild-type and *Has3−/−* mice (Fig. 2d). Increased macrovesicular steatosis was more abundant in the livers from the *Has3−/−* mice compared to the wild-type mice 24 h after CCl_4_. Consistent with increased plasma ALT values, hepatic architecture was more severely disrupted in the *Has3−/−* mice 48 h after CCl_4_ exposure. Fewer hepatocyte nuclei and more hemorrhage were present in the necrotic areas of the *Has3−/−* mice compared to the wild-type mice. Seventy-two hours after CCl_4_, robust cellular infiltration was found in the pericentral areas of the wild-type mice; this infiltration was reduced in the livers from the *Has3−/−* mice and was associated with more eosinophilic staining in the pericentral areas (Fig. 2d). Ninety-six hours after CCl_4_ exposure, liver histology was largely back to normal and not different between genotypes (data not shown).

**Fig. 3:**
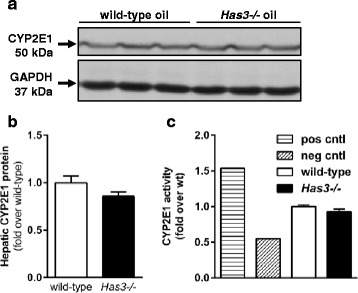
CYP2E1 content and activity in wild-type and *Has3−/−* mice. **a** Representative CYP2E1 immunoblot using samples from olive oil-treated wild-type and *Has3−/−* mice. GAPDH was used as a loading control. **b** Semi-quantification of hepatic CYP2E1 content after normalization to GAPDH. Data are expressed as fold change over wild-type mice (*n* = 6 each genotype). **c** Hepatic CYP2E1 activity assay in microsomes isolated from the wild-type and *Has3−/−* mice at baseline (before CCl_4_ exposure) (*n* = 6 mice per experimental group). The data are expressed as fold change over wild-type. *Pos cntl*, microsomes prepared from a single mouse fed an ethanol-containing diet for 5 weeks (ethanol is known to increase CYP2E1). *Neg cntl*, microsomes prepared from a single mouse exposed to CCl_4_ and euthanized 24 h later (CCl_4_ consumptively depletes CYP2E1)

CCl_4_ must be bioactivated to reactive metabolites to induce hepatotoxicity [23]. To ensure that increased liver injury in *Has3−/−* mice was not due to differences in CCl_4_ bioactivation, we measured cytochrome P450 2E1 (CYP2E1) protein (Fig. 3a, b) and activity (Fig. 3c) levels; there was no difference in these parameters between genotypes. Surprisingly, in contrast to the pathogenic role HAS3 plays in injury in other animal models of human disease, our data suggest that HAS3 plays a protective role in the liver after acute CCl_4_ exposure.

### Hepatic inflammation after acute CCl_4_ exposure in wild-type and *Has3−/−* mice

**Fig. 4:**
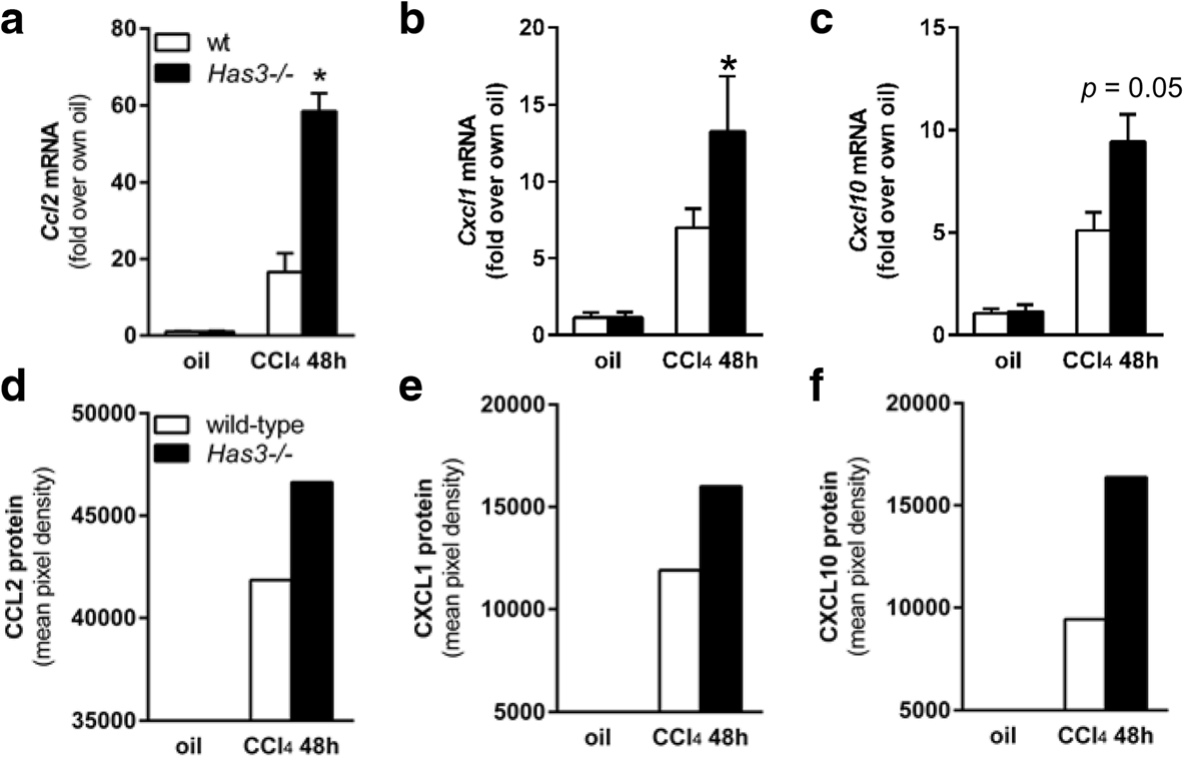
Hepatic and plasma chemokine content in wild-type and *Has3−/−* mice. Forty-eight hours after CCl_4_ exposure, the mice were euthanized and the livers were collected to determine the relative amounts of **a** *Ccl2*, **b** *Cxcl1*, and **c** *Cxcl10* transcripts in both genotypes. Here and throughout the manuscript, data were calculated using the 2^−ΔΔCt^ method after normalization to 18S and are expressed as fold change over each genotype’s own baseline (oil). Plasma isolated from blood collected at the same time points was used to determine the relative amounts of CCL2 (**d**), CXCL1 (**e**), and CXCL10 (**f**) peptides using a protein array. For this assay, plasma samples were pooled per experimental group (*n* = 6–8 mice). The *bar graphs* in **d**–**f** were created after semi-quantification of chemokine spot density from the array after densitometric analysis (spots not shown). *P < 0.05 compared between genotypes at the same time point

We next investigated the impact HAS3-deficiency on hepatic inflammation. While inflammatory markers increased in the livers from both strains of mice after CCl_4_ exposure, these markers tended to be greater in the *Has3−/−* mice compared to the wild-type mice. Specifically, using real-time PCR, we found hepatic *Ccl2*, *Cxcl1*, and *Cxcl10* were increased in the *Has3−/−* mice (Fig. 4a–c). None of these transcripts were different between genotypes at other time points (data not shown). Consistently, using a cytokine protein array, we found that plasma CCL2, CXCL1, and CXCL10 chemokine proteins were also increased in the *Has3−/−* mice (Fig. 4d–f). High *Cd11b* expression is associated with pro-inflammatory, infiltrating macrophages after acute liver injury [24]. While precise evaluation of the inflammatory infiltrate is outside the goal of this manuscript, we were able to detect an increase in hepatic accumulation of *Cd11b* transcripts in *Has3-/-* mice which approached significance (Additional file 1).

### Effect of *Has3*-deficiency on hepatic stellate cell activation and fibrosis after acute and chronic CCl_4_

**Fig. 5:**
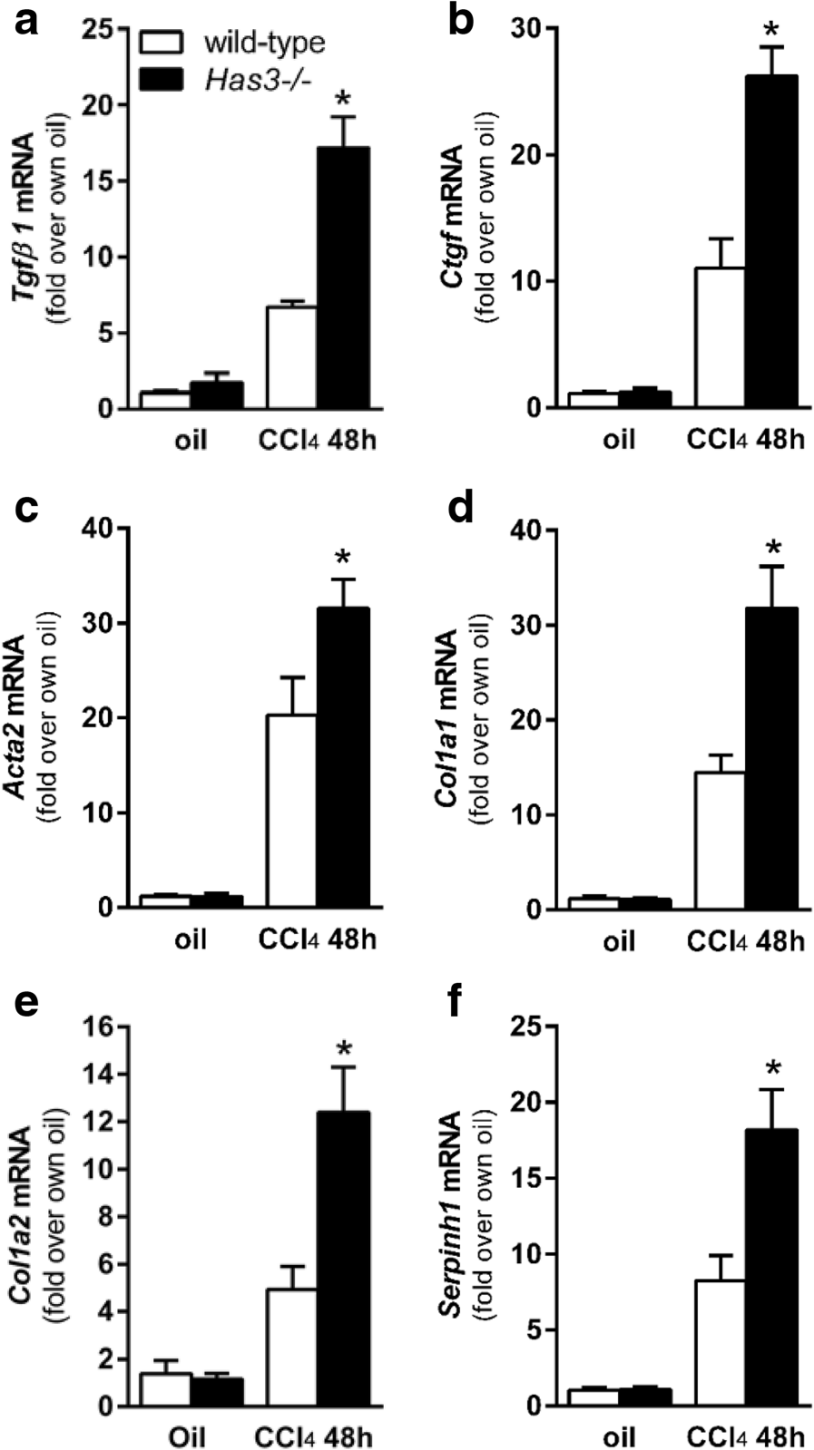
Hepatic fibrotic marker transcript accumulation after acute CCl_4_ exposure. The mice were exposed to CCl_4_ and euthanized 48 h later. Control animals received an olive oil (oil) injection. Hepatic **a**. *Tgfβ1*, **b** *Ctgf*, **c** *Acta2*, **d** *Col1a1*, **e** *Col1a2*, and **f** *Serpinh1* were measured using real-time PCR. The data are expressed as fold change over each strain’s olive oil-treated control after normalizing for input cDNA using 18S (*n* = 6–8 mice per experimental group). *P < 0.05 compared between genotypes at the same time point

Although acute CCl_4_ exposure does not cause fibrosis, it does induce activation of hepatic stellate cells (HSC), cells integral to hepatic wound healing through, in part, matrix remodeling and matrix synthesis. Because liver injury and inflammation were increased in the *Has3−/−* mice after acute CCl_4_ exposure, we predicted that the wound healing response would also be increased. After acute CCl_4_ exposure, markers associated with HSC activation were increased in both mouse strains. However, these markers were greater in the livers from the *Has3−/−* mice 48 h after CCl_4_. Specifically, using real-time PCR, we found that hepatic pro-fibrotic growth factor transcript accumulation (transforming growth factor (*Tgf*) *β1* and connective tissue growth factor (*Ctgf*)) were increased in the livers from the *Has3−/−* mice (Fig. 5a, b). Consistent with a role for these mediators in HSC activation, hepatic α smooth muscle actin (αSMA, *Acta2*), type I collagen (*Col1a1*, *Col1a2*), and heat shock protein 47 (*Serpinh1*), the collagen-specific chaperone, were increased 48 h after CCl_4_ exposure. These transcripts were each increased further in the HAS3-deficient mice (Fig. 5c–f). Collectively, these data suggest that increased injury and inflammation in the *Has3−/−* mice induces a more robust wound healing response in the liver after acute CCl_4_.

**Fig. 6:**
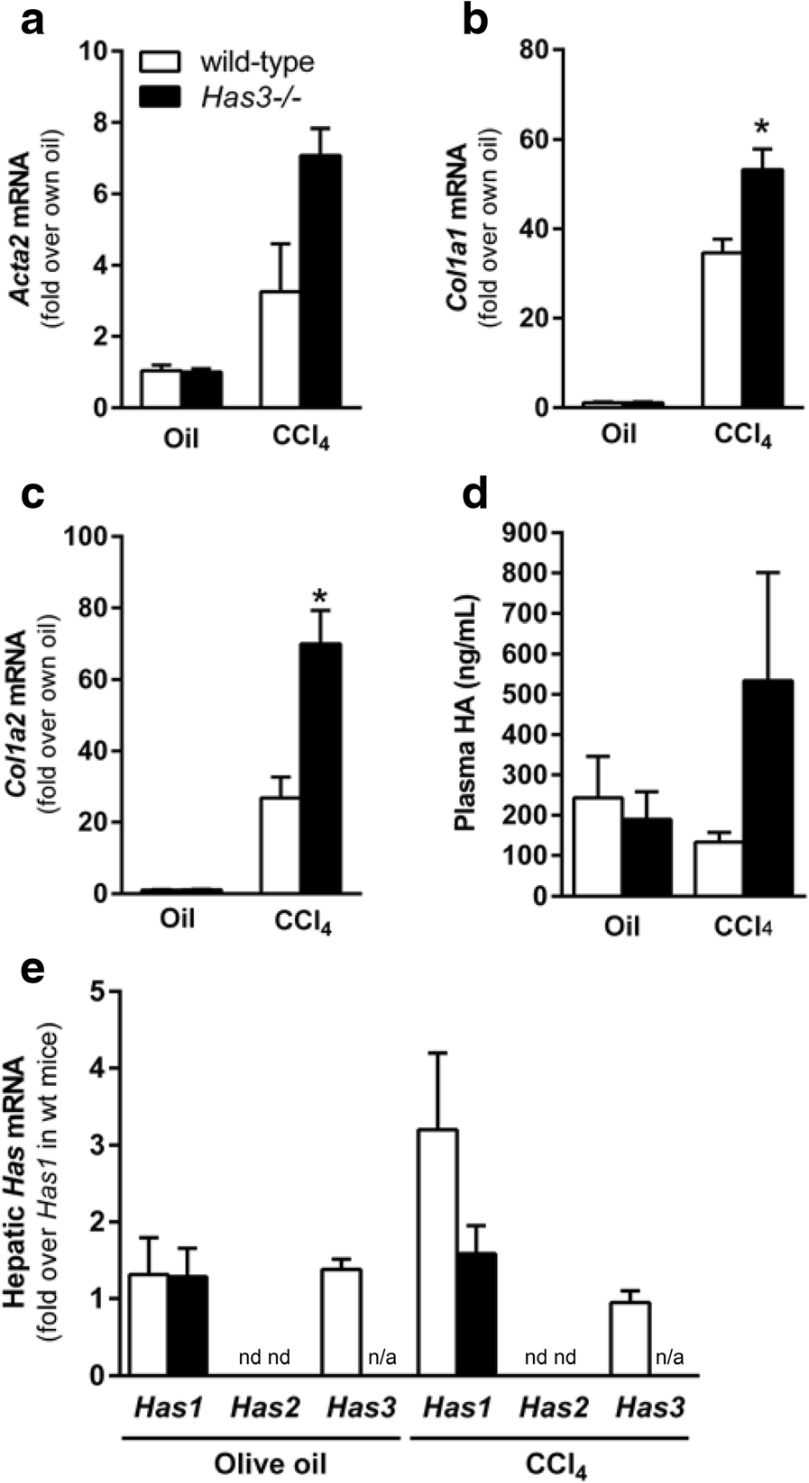
Hepatic fibrotic marker transcript accumulation and plasma HA levels after chronic CCl_4_ exposure. The mice were exposed to CCl_4_ for 5 weeks, twice per week, and were euthanized 72 h after the final CCl_4_ exposure. Control animals received olive oil (oil) injections. Hepatic **a** *Acta2*, **b** *Col1a1*, and **c** *Col1a2* transcript accumulation was determined using real-time PCR. The data are expressed as fold change over each strain’s olive oil-treated control after normalization to 18S. **d** HA concentration was determined using an HA ELISA-like assay (see the “Methods” section for details). **e** Hepatic *Has* enzyme transcript levels in wild-type and *Has3−/−* mice. Data were normalized to *Has1* levels in wild-type mice at baseline. *N* = 5–6 mice per experimental group. *nd* not detected, i.e., transcripts were below the level of detection, *n/a* not applicable. *P < 0.05 compared between genotypes

**Fig. 7:**
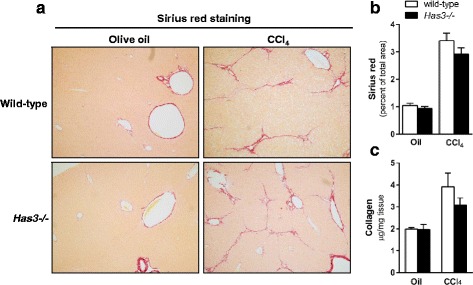
Liver fibrosis in wild-type and *Has3−/−* mice after chronic CCl_4_ exposure. The mice were exposed to CCl_4_ for 5 weeks, twice per week, and were euthanized 72 h after the final CCl_4_ exposure. Control animals received olive oil (oil) injections. **a** Sirius red was used to localize extracellular matrix accumulation in the livers from the control and CCl_4_-treated mice (*red bands* around vascular units and extending from and connecting neighboring central veins, ×100 magnification). **b** Semi-quantification of Sirius red staining expressed as a percent of total tissue area. Similar quantification of Sirius red staining intensity was also performed, and no differences were observed between groups (data not shown). **c** Hepatic hydroxyproline concentration was determined using a biochemical assay from which approximate collagen content was calculated (see the “Methods” for further details). *N* = 5–6 mice per experimental group

To evaluate the impact HAS3-deficiency had on frank fibrosis, we administered CCl_4_ twice per week for 5 weeks to wild-type and *Has3−/−* mice. Real-time PCR data revealed no difference in actin alpha 2, smooth muscle, aorta (*Acta2*) (αSMA) transcripts after chronic CCl_4_ exposure (Fig. 6a). *Col1a1 and Col1a2* transcript accumulation was also induced in both strains and was increased in the *Has3−/−* mice (Fig. 6b, c). Interestingly, and in contrast to HA levels after acute CCl_4_, HA levels were not reduced in the *Has3−/−* mice at baseline or 72 h after CCl_4_. Although not significant, a trend to an increase in plasma HA was observed in the *Has3−/−* mice after chronic CCl_4_ exposure (Fig. 6d). Has enzyme transcript levels did not account for this difference in plasma HA concentration (Fig. 6e). To evaluate actual hepatic content of ECM proteins, liver sections from each mouse were stained using Sirius red. Semi-quantification of Sirius red staining demonstrated established fibrosis in both strains of mice (Fig. 7a). However, after morphometry to quantify Sirius red-positive staining, there was no difference in ECM accumulation between genotypes (Fig. 7b). Consistently, collagen content, measured biochemically, was not different between the wild-type and *Has3−/−* mice (Fig. 7c).

### Evaluation of matrix remodeling in wild-type and *Has3−/−* mice

**Fig. 8:**
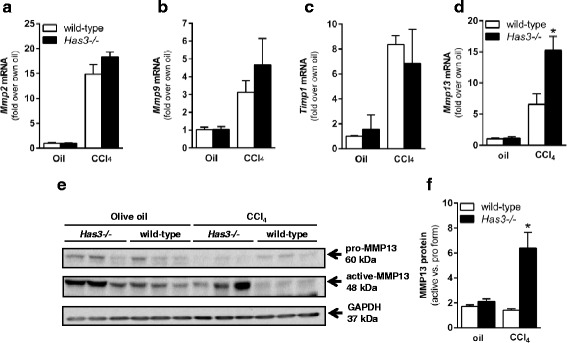
Hepatic *Mmp* and *Timp* content in the livers from wild-type and *Has3−/−* mice. The mice were exposed to CCl_4_ for 5 weeks, twice per week, then were euthanized 72 h after the final CCl_4_ exposure. Control animals received olive oil (oil) injections. Hepatic **a** *Mmp2*, **b** *Mmp9*, **c** *Timp1* and **d** *Mmp13* transcript accumulation was evaluated using real-time PCR. Data are expressed as fold change over each strain’s baseline (oil) after normalization to 18S. **e** Representative immunoblot of hepatic pro and active MMP13 protein. GAPDH is used to demonstrate equal loading. **f** Semi-quantification MMP13 band densities expressed as relative amount of active MMP13 relative to pro MMP13. *N* = 5–6 mice per experimental group. *P < 0.05 compared between genotypes

**Fig. 9:**
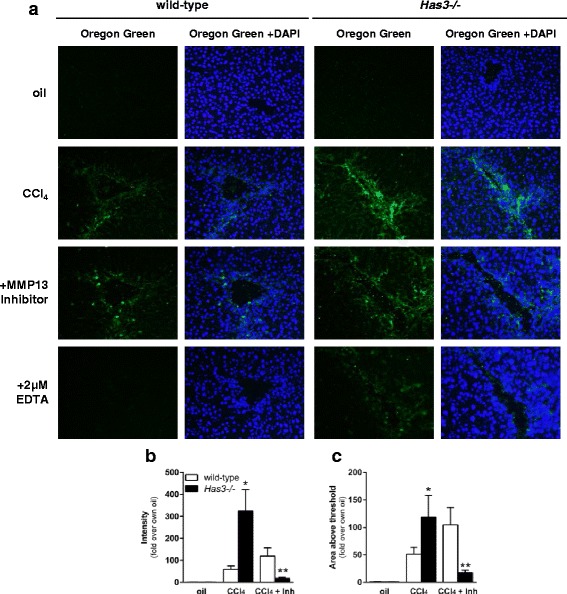
Matrix metabolism in wild-type and *Has3−/−* mice after chronic CCl_4_ exposure. Mice were exposed to CCl_4_ for 5 weeks, twice per week, then were euthanized 72 h after the final CCl_4_ exposure. Control animals received olive oil (oil) injections. **a** In situ zymography in the wild-type and *Has3−/−* mice. In the *first column* under each genotype, green fluorescence (Oregon Green 488) reveals matrix-degrading activity. In the *second column*, images were captured after DAPI staining to visualize hepatic nuclei. The *third column* contains merged images (green fluorescence and DAPI). **a** MMP13-specific inhibitor (Inh) was used to attenuate MMP13-specific matrix-degrading activity; EDTA was used to block all MMP activity. **b** Intensity of green fluorescence and **c** area of green fluorescence above threshold were determined using ImageJ. *N* = 5–6 mice per experimental group. *P < 0.05 compared between genotypes; **P < 0.05 when compared to untreated (no Mmp13 inhibitor) and to wild-type treated with MMP13 inhibitor

Faced with an apparent paradox (i.e., increased liver injury, inflammation, and fibrotic transcript accumulation after acute liver injury but equivalent hepatic ECM content after chronic liver injury), we hypothesized that the *Has3−/−* mice had an increased ability to remodel their hepatic ECM after chronic CCl_4_ exposure; increased ability to degrade the ECM could be responsible for the equivalent ECM content in livers from both mouse strains. Using real-time PCR, we evaluated hepatic accumulation of matrix metalloproteinase (*Mmp*) and tissue inhibitor of matrix metalloproteinase (*Timp*) transcripts after chronic CCl_4_. While there was no difference in *Mmp2*, *Mmp9*, or *Timp1* (Fig. 8a–c), *Mmp13* transcripts were increased in the livers from the *Has3−/−* mice; there was no difference between baseline *Mmp13* transcript levels between genotypes (Fig. 8d). We used immunoblotting to further evaluate hepatic MMP13. We found that, under basal conditions, the livers from the *Has3−/−* and wild-type mice had a substantial active form of the MMP13 enzyme; the ratio between active and pro MMP13 was not different between genotypes (Fig. 8e, f). This likely reflects normal matrix remodeling. However, after chronic CCl_4_ exposure, only the *Has3−/−* mice had an increase in the ratio of active MMP13 to pro MMP13. To determine if these changes in hepatic MMP13 resulted in increased matrix remodeling, we performed in situ zymography, in vitro, using dye-quenched gelatin as a substrate [25]. In this technique, location, area, and intensity of a matrix-degrading activity is visualized by the presence of green fluorescence. This technique revealed matrix metabolism in the fibrotic septae in the liver sections from the wild-type and HAS3-deficient mice (Fig. 9a). However, the intensity and total area of fluorescence (i.e., matrix remodeling) was threefold greater in the *Has3−/−* mice (Fig. 9b, c). Importantly, when incubated with a MMP13-specific inhibitor, matrix remodeling was reduced in the livers from the *Has3−/−* mice; the inhibitor did not suppress matrix metabolism in the livers from the wild-type mice. Therefore, even though the *Has3−/−* mice exhibited worse liver injury, inflammation and pro-fibrotic gene expression after acute CCl_4_, this did not precipitate increased fibrosis after chronic CCl_4_ exposure. Instead, the *Has3−/−* mice exhibited an increased capacity to remodel the hepatic ECM during fibrosis limiting fibrotic disease.

## Discussion

In this manuscript, we provide the first evidence to suggest that HAS3, and presumably HA produced by HAS3, plays differential roles after acute and chronic liver injury. Specifically, HAS3 protects the liver after acute liver injury by dampening hepatic inflammation. Our data suggest that this protective effect may be due to the total amount and relative proportions of critical molecular weight HA pools. In addition, the HAS-deficient mice exhibited an enhanced ability to remodel the ECM after chronic CCl_4_, and this was dependent on MMP13. The presence of more, lower molecular weight HA fragments in *Has3−/−* mice may have driven the enhanced MMP13 response as shown previously [26]. Enhanced matrix remodeling prevented the increase in fibrosis we predicted based on hepatic fibrotic gene transcript accumulation in *Has3−/−* mice after acute and chronic CCl_4_ exposure. Therefore, HAS3-mediated HA production is pathologic in chronic liver injury. This dichotomous relationship between the roles for HAS3 after acute or chronic liver injury likely reflects important differences between where in the liver wound healing continuum one looks.

Despite a well-appreciated role for plasma HA as a hepatic function biomarker [18, 27, 28], few studies have explored the role HA plays in pathogenesis or resolution of liver disease. It is known that HSC can synthesize HA and do so in response to partial hepatectomy, a surgical model of liver regeneration [29]. In addition, treating mice with HMW-HA (780, 900, or 1200 kDa), but not LMW-HA (250 and 470 kDa), can prevent T cell mediated liver injury (concanavalin A and galactosamine/lipopolysaccharide models), and this occurs through a reduction in pro-inflammatory cytokines, including tumor necrosis factor α, interferon gamma, macrophage inflammatory protein 2 (MIP2), and interleukin 4 [30]. Administration of human umbilical vein HA (HMW-HA) also exerts some protection against CCl_4_-induced liver injury, lipid peroxidation, and fibrosis in rats when used in combination with chondroitin sulfate, but not when either molecule was used alone [31]. Collectively, these studies, as well as our study, suggest that HA can exhibit a protective effect in animal models of acute liver disease.

While no one has yet explored the effect of HAS enzyme deficiency in animal models of liver injury, investigators have evaluated the impact HAS3 deficiency has in other animal models of human disease; these studies largely support a pathogenic role for HAS3. For example, in the lung, Bai et al. demonstrated that high tidal volume, ventilator-induced pulmonary MIP2 production, and neutrophil infiltration are robust in wild-type mice, but suppressed in mice deficient in HAS3 [14]. The increase in inflammation in wild-type mice is associated with increased pulmonary accumulation of HA favoring lower molecular weight species (178–370 kDa); this was not observed in *Has3−/−* mice. Likewise, Kessler et al. demonstrated that HAS3-null mice are protected from dextran sodium sulfate (DSS)-induced colitis when compared to wild-type mice [16]. In the absence of HAS3, HA deposition and leukocyte infiltration are profoundly attenuated and associated with reduced weight loss and pro-inflammatory cytokine production after DSS exposure. In both of these studies, and in contrast to our study, HAS3 drives tissue inflammation.

The specific mechanisms behind pathogenic function of HAS3 in the lung and in the gut remain controversial. One hypothesis is that because HAS3 can make smaller HA polymers compared to HAS1 and HAS2 [8], perhaps HAS3-synthesized HA is small enough to be pro-inflammatory on its own. Conversely, perhaps LMW-HA synthesized by HAS3 is not directly pro-inflammatory but because it is smaller initially, it is more rapidly degraded into HA species which are pro-inflammatory. Another possible explanation is that HAS enzymes are differentially expressed in various tissues, cell types, or in response to different stimuli. Therefore, loss of one enzyme could profoundly influence the nature of the inflammatory response to injury in a tissue or cell-type-specific fashion [7]. Therefore HAS3’s differential effect, pathologic vs protective depending on the animal model or in acute vs chronic disease, should be explored further by evaluating in which cell types HAS3 is expressed as well as by elucidating the local production and turnover of HA after tissue injury. Indeed, in the DSS-induced colitis model, HAS3 expression is predominantly localized to the endothelial cells found in gut microvessels where it produces a leukocyte-adhesive HA and contributes to inflammation [32]. Further exploration of cell-type-specific hepatic HAS enzyme expression, hepatic HA accumulation, and hepatic HA molecular weight distribution during liver disease pathogenesis, progression, or resolution is required to understand the differences between our study and the other studies discussed above.

In this study, we observed increases in hepatic mRNA and plasma protein levels for chemokines and CD11b, a marker associated with monocyte recruitment to the liver [24] associated with tissue inflammation. These chemokines can attract a number of cells involved in innate an adaptive immune responses to tissue injury including monocytes/macrophages (CCL2, CXCL10), neutrophils (CXCL1), natural killer cells (CXCL10), dendritic cells (CCL2, CXCL12), and T cells (CCL2, CXCL10). We believe that chemokine production is at a critical nexus between the promotion of liver injury and, paradoxically, resolution of fibrosis. Indeed, while CYP2E1-mediated bioactivation is required for CCl_4_’s hepatotoxic effects [33], macrophages and macrophage recruitment also contribute to liver injury in this model. Specifically, if macrophages are depleted using gadolinium chloride, liver injury after CCl_4_ exposure is reduced [34]. Similarly, the mice deficient in CCL2 or the CCL2 receptor, CCR2, exhibit reduced plasma ALT activities, intrahepatic inflammatory cytokine expression, and macrophage recruitment after CCl_4_ exposure [35, 36]. Therefore, increased inflammatory macrophage recruitment in response to increased chemokine production in *Has3−/−* mice could contribute to increased injury after CCl_4_ exposure.

In addition to exacerbating liver injury and inflammation, hepatic macrophages and macrophage recruitment are also critical determinants of fibrosis resolution. For example, depleting macrophages with diphtheria toxin using a CD11b-diphtheria toxin receptor transgenic mouse attenuates matrix degradation in fibrotic liver [37]. Likewise, CCl_4_-induced fibrosis resolution is reduced when macrophage recruitment is prevented in CCR2-deficient mice [36]. Therefore, enhanced chemokine production in response to acute CCl_4_ could facilitate better fibrosis resolution by recruiting more macrophages to the injured liver.

As described by others, MMP13 is critical for resolution of liver fibrosis in mice and is associated with a resolution-associated macrophage population called scar-associated macrophages (SAMs) [38]. MMP13 degrades fibrillar collagen and also exhibits gelatinase activity [39, 40]. Consistently, in mice with reduced hepatic SAMs, fibrosis resolution after cessation of CCl_4_ exposure is delayed and associated with reduced hepatic expression of *Mmp13* mRNA and active MMP13 enzyme [36, 41]. It is important to note, however, that global deletion of MMP13 reduces fibrosis after bile duct ligation (BDL). Uchinami et al. demonstrated that reduced liver injury and inflammation in MMP13-deficient mice was responsible for the diminished fibrotic response after BDL [42]. This suggests that the role MMP13 plays in different points in the wound healing response may be critical to its overall impact on fibrosis. In this study, we found increased *Mmp13* transcript accumulation, active form of MMP13 and MMP13 enzyme activity after chronic CCl_4_; we believe this contributed to the increased matrix remodeling in the *Has3−/−* mice. HA oligosaccharides are known inducers of MMP13 [26] It is tempting to speculate that increased HA levels found in the *Has3−/−* mice after chronic exposure to CCl_4_ may have contributed to the increase in MMP13 levels we found in this study. While beyond the scope of this manuscript, we are currently exploring macrophage phenotype in *Has3−/*− mice after chronic CCl_4_ and the response of macrophages to specific HA polymer sizes to more clearly understand the mechanism behind the observations made in this study.

## Conclusions

We have demonstrated that HAS3, or HA synthesized by HAS3, is a critical determinant of the divergent outcomes observed after acute and chronic liver injury in response to CCl_4_. Importantly, our data suggest that HA plays two roles in the liver, one in which it attenuates liver injury and inflammation and one in which it exacerbates fibrosis. Further work is required to understand this apparently dichotomous relationship between HA and acute vs chronic liver injury and leverage that information for the development of HA-targeted therapeutics in liver disease.

## Methods

### Reagents

Primary antibodies used include the following: CYP2E1 (Abcam, Cambridge, MA), MMP13 (clone LIPCO-IID1, Abcam), and glyceraldehyde 3-phosphate dehydrogenase (GAPDH) (Cell Signaling, Clone 14C10, Beverly, MA). A horseradish peroxidase (HRP)-conjugated goat-anti-rabbit secondary antibody (Abcam) was used for the primary antibodies listed above. Olive oil and carbon tetrachloride were purchased from Sigma-Aldrich (St. Louis, MO), Buprenex analgesic (buprenorphine HCl) was manufactured by Reckitt Benckiser Healthcare (UK, Ltd, Hull England) and distributed by Reckitt Benckiser Pharmaceuticals, Inc. (Richmond, VA). Anesthetics used were from the following sources: ketamine (Akorn, Inc, Decator, IL), xylazine (KetaVed, VedCO, Inc., St. Joseph, MO), and acepromazine (VedCO, Inc.). Pyrimidine-4,6-dicarboxylic acid, bis-(4-fluoro-3-methyl-benzylamide) was used as a selective MMP13 inhibitor (EMD Millipore/Calbiochem, Billerica, MA, catalog number #444283).

### Animal care

The animals were treated humanely and in accordance to protocols approved by the University of Kansas Medical Center’s (KUMC) Institutional Animal Care and Use Committee (IACUC). The mice were housed in ventilated cages on a 10/14-h light/dark cycle with access to standard mouse chow and water ad libitum. The *Has3−/−* mice were generated by Bai et al. through gene targeting, eliminating the catalytic site of the HAS3 enzyme [14]. Male *Has3−/−* mice (C57BL/6J background, confirmed by NNT genotyping) were bred at KUMC. Age-matched, wild-type (C57BL/6 J), male mice (acute CCl_4_ studies, see below) or female and male mice (chronic CCl_4_ studies) were purchased from Jackson Labs (Bar Harbor, ME) and used within 2 weeks of arrival at KUMC. There were no differences in disease pathogenesis parameters between male and female mice in chronic CCl_4_ studies. NNT genotyping confirmed that the *Has3−/−* mice and C57BL/6J mice were on the same (BL/6J) genetic background.

### Carbon tetrachloride exposure, tissue collection, and storage

In acute CCl_4_ studies, the mice were given a single intraperitoneal (i.p.) injection of CCl_4_ at a concentration of 0.4 mg/g body weight diluted 1:3 in olive oil. In the chronic studies, the mice were given two CCl_4_ injections per week for 5 weeks ramping up from 0.1 mg/g BW (one injection) to 0.2 mg/g BW (one injection) to 0.4 mg/g BW (8 eight injections). Subcutaneous administration of an analgesic (buprenorphine) preceded each CCl_4_ injection by 10 min as done previously [43] and on recommendation by the KUMC IACUC. The control mice received analgesic and olive oil injection(s). Twenty-four, 48, 72, or 96 h (acute) or 72 h (chronic) post CCl_4_, the mice were anesthetized using a cocktail of ketamine (200 mg/kg), xylazine (40 mg/kg), and acepromazine (20 mg/kg). Blood was collected from the inferior vena cava into EDTA and aprotinin-containing tubes and placed on ice. After the blood was collected, the diaphragm, superior vena cava, and aorta were cut euthanizing the mouse. After euthanasia, a hepatectomy was performed. The liver was divided into several pieces while on an ice-chilled piece of glass: the small half of the median lobe was cut into three pieces and placed into 2-mL tubes with 1.5 mL of RNAlater (Life Technologies, Grand Island, NY) stored at room temperature for 5 min, at 4 °C for 18 h and then transferred to −20 °C until use. A portion of the large half of the median lobe was embedded in Optimal Cutting Temperature (OCT) medium, incubated on a bed of frozen isopentane until the OCT was opaque and then stored at −80 °C. The largest lobe of the liver (left lobe) was cut into several slices, and some of which were used for Western blot analysis (snap frozen in liquid nitrogen, stored at −80 °C) or fixed in formalin and later embedded in paraffin for histological analysis. The right lobe was snap frozen in liquid nitrogen and then stored at −80 °C for triglyceride quantification. All remaining liver tissue was snap frozen and archived at −80 °C; CYP2E1 activity assays were performed using pieces from the left lobe. The blood was centrifuged at 10,000×*g* for 3.5 min. Plasma was separated into two aliquots and frozen at −80 °C until use.

### Liver injury and hepatic triglyceride content determination

Plasma alanine aminotransferase (ALT) activity was determined using a commercially available enzymatic assay (Sekisui Diagnostics, Exton, PA) according to the manufacturer’s instructions. Activity was calculated using the extinction coefficient method. For triglyceride measurement, the livers were digested with 3 M KOH in 65 % ethanol for 1 h at 70 °C and vortexed every 20 min to aid in tissue disruption. Twenty-four hours later, samples were diluted 1:5 in 2 M tris pH 7.5, 10 μL was added to triglyceride GPO reagent (Trinder method), and absorbances were read at 500 nm. A standard curve created using a GPO standard was used to calculate total hepatic triglyceride content (Pointe Scientific, Canton, MI).

### Histological analysis

Formalin-fixed, paraffin-embedded sections were cut (5 μM) and stained with hematoxylin and eosin. Micrographs were taken at ×200 magnification using an Olympus BX51 microscope fitted with an Olympus DP71 camera (Olympus, Waltham, MA). DP Controller software was used to acquire images (Olympus, Waltham, MA). Three non-overlapping images per liver section were acquired and viewed by a blinded individual (MFO). Assessments were made on a scale from 0 to 4+ for the following parameters: cellular infiltrate, steatosis, necrosis, and hemorrhage.

### Immunoblotting

Liver lysates were prepared as described [43], and samples were resolved on 10 % SDS-PAGE gels. Proteins were transferred to PVDF membranes, blocked in 5 % BSA, and then probed for proteins of interest overnight at 4 °C with agitation. HRP-conjugated secondary antibodies were used, and after incubation in an Enhanced Chemiluminescent substrate (GE Healthcare, Piscataway, NJ), luminescence was captured using radiographic film. Quantification of band density was achieved using ImageJ. Data were normalized to a housekeeping gene (GAPDH).

### CYP2E1 activity assay

Liver microsomes were prepared by homogenizing 100–150 mg of frozen liver tissue in 1 mL of cold PBS with a loose fitting dounce homogenizer. After centrifugation at 9000×*g* for 15 min, the fat layer was removed from the surface of the liquid and 10 mL of cold PBS was added and the homogenate was centrifuged at 105,000×g for 1 h at 4 °C. The pellet was resuspended in 0.15 M KCl, and the total protein concentration was determined by BCA assay (Life Techologies/Pierce, Grand Island, NY). Four microliters of 10 mM p-nitrophenol, and 10 μL phosphate buffer (4 mL 1 M K2HPO4 + 1 mL 1 M KH2PO4 pH 7.4) were added to 30 μg of protein; water was added to bring the total volume to 100 μL. Ten microliters of freshly prepared NADPH (10 nM) was then added, and the samples were incubated at 37 °C in a water bath for 1 h. Following incubation, 30 μL of 20 % trichloroacetic acid was added, and the samples were vortexed, then centrifuged 10,000×g for 10 min. One hundred microliters of supernatant was added to 10 μL 10 N NaOH, and absorbance was determined at 510 nm. CYP2E1 activity was calculated using the extinction coefficient of 9.53 × 10^5^ M^−1^ cm^−1^, normalized to protein concentration, and expressed as fold change over wild-type, oil-exposed (control) mice.

### RNA isolation, cDNA synthesis, and real-time PCR

RNAlater-stabilized liver pieces (20–30 mg) were homogenized (FastPrep 24, MP Biomedicals, Solon, OH) in RLT buffer (RNeasy Mini Kit, Qiagen, Valencia, CA) with 10 μL of β-mercaptoethanol per mL of RLT. RNA was then isolated using an RNeasy Mini Kit; 4 μg of RNA was reverse transcribed into complementary DNA (cDNA) using a Retroscript kit (Life Technologies/Ambion, Grand Island, NY). SYBR green (BioRad Universal Super Mix,) was used for real-time PCR in a BioRad CFX384 real-time PCR machine, and results were calculated using 2^−ΔΔCt^ method. Data were expressed as fold change over olive oil-treated mice within a genotype. Primers utilized in this study are found in Table 2; 18S was used as the housekeeping gene and did not differ between genotypes or time points after CCl_4_. Sequence sources are noted in the table, most of which were obtained from the PrimerBank (http://pga.mgh.harvard.edu/primerbank/) [44–46].

**Table 2:** Primers used for real-time PCR transcript analysis

Gene name	Protein	Gen bank acc #	Sequence source	Forward primer	Reverse primer
*Has1*	Has1	NM_008215	PrimerBank: 6680169a1	GGCGAGCACTCACGATCATC	AGGAGTCCATAGCGATCTGAAG
*Has2*	Has2	NM_008216	Matrix Biol. 30(2):126–134, 2011	GGTCCAAGTGCCTTACTGAAAC	TGTACAGCCACTCTCGGAAGTA
*Has3*	Has3	NM_008217	PrimerBank: 6680173a1	GTGGGCACCAGTCTGTTTG	CCACTGAACGCGACCTCTG
*Ccl2*	CCL2/MCP1	NM_011333	Immunol. Cell Biol. 89:716, 2011	AGGTCCCTGTCATGCTTCTG	TCTGGACCCATTCCTTCTTG
*Cxcl1*	CXCL1/GRO1/KC	NM_008176	PrimerBank: 229577225c1	ACTGCACCCAAACCGAAGTC	TGGGGACACCTTTTAGCATCTT
*Cxcl10*	CXCL10/IP10	NM_021274	PrimerBank: 10946576a1	CCAAGTGCTGCCGTCATTTTC	GGCTCGCAGGGATGATTTCAA
*Cd11b*	CD11b	NM_001082960	PrimerBank: 132626288c3	GGGAGGACAAAAACTGCCTCA	ACAACTAGGATCTTCGCAGCAT
*Tgfb1*	TGFβ1	NM_011577	PrimerBank: 6755774b1	AGCTGGTGAAACGGAAGCG	GCGAGCCTTAGTTTGGACAGG
*Ctgf*	CTGF	NM_010217	PrimerBank: 6753878a1	GGGCCTCTTCTGCGATTTC	ATCCAGGCAAGTGCATTGGTA
*Acta2*	αSMA	NM_007392	PrimerBank: 31982518b1	CCCAGACATCAGGGAGTAATGG	TCTATCGGATACTTCAGCGTCA
*Col1a1*	COL1A1	NM_007742	World J. Gastroenterol. 4(12):356, 2012	ATGTTCAGCTTTGTGGACCTC	CAGAAAGCACAGCACTCGC
*Col1a2*	COL1A2	NM_007743	Am. J. Pathol. 176(6):2743, 2010	GGTGAGCCTGGTCAAACGG	ACTGTGTCCTTTCACGCCTTT
*Serpinh1*	HSP47	NM_009825	PrimerBank: 6753304a1	GCCGAGGTGAAGAAACCCC	CATCGCCTGATATAGGCTGAAG
*Mmp2*	MMP2	NM_008610	PrimerBank: 47271505b1	GATGTCGCCCCTAAAACAGAC	CAGCCATAGAAAGTGTTCAGGT
*Mmp9*	MMP9	NM_013599	PrimerBank: 31560795b2	GGACCCGAAGCGGACATTG	GAAGGGATACCCGTCTCCGT
*Mmp13*	MMP13	NM_008607	PrimerBank: 291463259b1	TGTTTGCAGAGCACTACTTGAA	CAGTCACCTCTAAGCCAAAGAAA
*Timp1*	TIMP1	NM_011593	PrimerBank: 6755795a1	GCAACTCGGACCTGGTCATAA	CGGCCCGTGATGAGAAACT

### Cytokine protein array

Plasma cytokine protein content was measured using a Proteome Profiler: Mouse Cytokine Array Panel A (R&D Systems) to evaluate inflammatory mediators present in pooled plasma samples from the mice exposed to CCl_4_ and euthanized 48 h later or in animals exposed to olive oil (control). In brief, pooled plasma samples (*n* = 6–8 individual mice per group) were applied to the provided membrane impregnated with capture antibodies. Streptavidin-HRP-conjugated secondary antibodies were then applied, and chemiluminescent technology was used to detect proteins captured by the array. All arrays were done on the same day with the same exposure time. NIH ImageJ was used to semi-quantify pixel density of resultant cytokine-positive areas recorded using radiographic film.

### Size-selective HA fractionation and quantification

Quantikine ELISA for HA (R&D Systems, Minneapolis, MN) was performed per manufacturer’s instructions to determine the total HA content in plasma. Because HA is a carbohydrate, the ELISA did not utilize capture and detection antibodies. Instead, it utilized HA-binding proteins (HABP). The wells of a 96-well plate were coated with a capture HABP by the manufacturer. A biotinylated HABP was utilized as a detection reagent. We refer to this modified ELISA as an “ELISA-like assay” in this study. Determination of a size-specific HA fraction in plasma was done as described previously [47], with modification. Briefly, plasma was pooled (*n* = 4–8 mice per time point) and digested 1:1 with 10× Proteinase K solution at 60 °C for 4 h. Molecular weight cut-off columns (Centrisart, 100 and 300 kDa, Sartorius, Goettingen, Germany) were used to isolate HA with molecular weights less than 100 kDa, between 100 and 300 kDa and greater than 300 kDa from the wild-type and *Has3−/−* mice.

### Sirius red staining, image acquisition and data collection

Formalin-fixed, paraffin-embedded liver tissue sections were incubated at 60 °C for 20 min, deparaffinized in SafeClear (xylene substitute, protocol II), and rehydrated in a graded series of ethanol. Slides were then immersed in 0.1 % picrosirius red solution (Direct Red 80, in saturated picric acid) for 1 h at room temperature. Acidified water (0.5 % glacial acetic acid in water) was used to wash the slides three times for 5 min each. The slides were dehydrated in a reverse series of graded ethanol and fresh SafeClear solutions, mounted using Permount and glass coverslips and allowed to dry. An Olympus BX51 microscope and Olympus DP71 camera were used to acquire images at 100× magnification using DP Controller software (Olympus, Waltham, MA). Five non-overlapping images per liver section were acquired. One liver section per mouse in each experimental group was photographed (*n* = 5–6 mice per group). ImageJ was used to quantify the area of positive staining above an arbitrary threshold which remained constant for all images.

### Hydroxyproline quantification and calculation of collagen content

Collagen content was determined using the hydroxyproline assay as described by Reddy and Enwemeka [48]. Briefly, 10 mg of liver tissue was digested in water and 12.1 N HCl at 120 °C for 3 h using a dry bath incubator. The samples were vortexed every 30 min during this incubation period. The samples were centrifuged 10,000×*g* for 10 min after the incubation was complete. Ten microliters of the resulting supernatant was placed, in duplicate, into wells of a 96-well plate. Hydroxyproline standards (0–1 μg/well) were also added to the plate, in duplicate. One hundred microliters of chloramine-T solution was added to each well and incubated for 25 min at room temperature, after which 100 μL of Ehrlich’s solution was added to each well and incubated further for 35 min at 60 °C in a dry heat incubator. Absorbances were measured at 550 nm, and the concentration of hydroxyproline was calculated from the standard curve. Collagen content was estimated by dividing the hydroxyproline concentration by 12.5 %.

### In situ zymography, image acquisition and data collection

Frozen tissue sections (7 μm) were taken from −80 °C and immediately incubated with developing buffer (100 mM Tris, pH 7.4, 100 mM NaCl, 5 mM CaCl_2_, 0.05 % Brij-35, 0.25 mM PMSF) containing 0.1 mg/mL Oregon green 488, dye-quenched (DQ) gelatin (Life Technologies/Molecular Probes, Grand Island, NY). To a second set of sections, an MMP13 inhibitor (EMD Millipore/Calbiochem, Billerica, MA, catalog number #444283) was added. This MMP13 inhibitor is also known as pyrimidine-4,6-dicarboxylic acid, bis-(4-fluoro-3-methyl-benzylamide), a non-zinc-chelating inhibitor of MMP13 [49]. It works by binding to the catalytic domain of the enzyme, preventing its activity. The slides were incubated in a humid chamber at 37 °C for 16–18 h. After this incubation, 4′,6-diamidino-2-phenylindole (DAPI) mounting medium was used as a nuclear counterstain and aqueous mounting medium, and then sealed using clear nail polish. An Olympus BX51 microscope with an Olympus BH2RFLT3 burner, Olympus DP71 camera, and DP Controller software were used to capture three non-overlapping images from each tissue section at 200× magnification. The exposure time was chosen to minimize autofluorescence in control liver sections and used for each subsequent image. ImageJ was used to quantify area and intensity of the fluorescent signal generated by matrix metabolism.

### Statistics

Data were collected from several independent experiments. All results are presented as means ± SEM or individual values alone. Statistical significance was defined as *P* ≤ 0.05 and denoted with an asterisk (*). Student’s *t* test was used when comparing two data sets, and ANOVA was used when comparing more than two data sets and included a Tukey’s adjustment for multiple comparisons. Analyses were performed using SAS (Cary, NC), GraphPad Prism 6 (La Jolla, CA), or Excel (Microsoft, Redmond, WA).

## Additional file

Additional file 1: Hepatic *Cd11b* transcript analysis. Real-time PCR was utilized to determine hepatic accumulation of *Cd11b* transcripts in wild-type and *Has3−/−* mice at baseline (oil) or 48 h after CCl_4_ exposure. (PDF 23 kb)

## Abbreviations

*Acta2*: actin alpha 2, smooth muscle, aorta
ALT: alanine aminotransferase
CCl_4_: carbon tetrachloride
*Col1a1*: collagen, type 1, alpha 1
*Col1a2*: collagen, type 1, alpha 2
*Ctgf*: connective tissue growth factor
CYP2E1: cytochrome P450 2E1
DAPI: 4′,6-diamidino-2-phenylindole
DSS: dextran sodium sulfate
ECM: extracellular matrix
ELISA: enzyme-linked immunosorbent assay
HA: hyaluronan
HAS: hyaluronan synthase
HMW: high molecular weight
HSC: hepatic stellate cell
LMW: low molecular weight
MMP: matrix metalloproteinase
SAM: scar-associated macrophage
*Serpinh1*: serine (or cysteine) peptidase inhibitor, clade H, member 1
*Tgfβ*: transforming growth factor
*Timp*: tissue inhibitor of matrix metalloproteinase
αSMA: alpha smooth muscle actin
