# Supplementary figures and images for: Differential effects of hyaluronan synthase 3 deficiency after acute vs chronic liver injury in mice
Source: Fibrogenesis Tissue Repair. 2016 Mar 31;9:4. doi: 10.1186/s13069-016-0041-5 (PMC4818527; doi:10.1186/s13069-016-0041-5)

Additional File 1

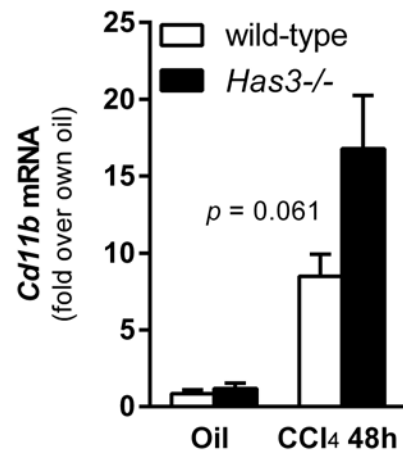

Supplement: Additional file 1: — Hepatic Cd11b transcript analysis. Real-time PCR was utilized to determine hepatic accumulation of Cd11b transcripts in wild-type and Has3−/− mice at baseline (oil) or 48 h after CCl4 exposure. (PDF 23 kb) [file 13069_2016_41_MOESM1_ESM.pdf]
